# Adequacy of Web-Based Activities as a Substitute for In-Person Activities for Older Persons During the COVID-19 Pandemic: Survey Study

**DOI:** 10.2196/25848

**Published:** 2021-01-22

**Authors:** Jiska Cohen-Mansfield, Aline Muff, Guy Meschiany, Shahar Lev-Ari

**Affiliations:** 1 Department of Health Promotion School of Public Health, Sackler Faculty of Medicine Tel Aviv University Tel Aviv Israel; 2 Minerva Center for the Interdisciplinary Study of End of Life Tel Aviv University Tel Aviv Israel; 3 Igor Orenstein Chair for the Study of Geriatrics Tel Aviv University Tel Aviv Israel

**Keywords:** web-based venues for older adults, social engagement, activities, boredom, technology barriers for seniors, COVID-19, pandemic, senior, elderly, older adult, online activity, engagement, activity, loneliness, isolation, effectiveness

## Abstract

**Background:**

Senior centers and other types of clubs provide activities for older adults to address boredom, social isolation, and loneliness. Due to the COVID-19 pandemic, most of these activities have been cancelled. A limited range of web-based activities have been offered as alternatives. However, the effectiveness of these web-based group activities for older adults has scarcely been researched.

**Objective:**

We aimed to understand the extent to which web-based activities for older adults provide an adequate substitute for in-person activities.

**Methods:**

In this telephone survey, we interviewed 105 older adults in Israel who had been offered the opportunity to participate in web-based activities after routine activities closed due to the COVID-19 pandemic. Of the total sample, 49/105 (46.7%) participated in the activities and 56/105 (53.3%) did not. We inquired about the respondents’ background characteristics, satisfaction with the activities, and reasons for participation or nonparticipation.

**Results:**

The respondents who participated in the web-based activities tended to be highly satisfied with at least some of them. They rated the enjoyment derived from the content of the activity as the most important motivator, followed by maintaining a routine and by enjoying the group and the presence of others. Over 50% of the participants (28/49, 57%) wished to continue with the exercise programming after the end of the COVID-19 pandemic, and 41% (20/49) wished to continue with the web-based lectures. Participants were more likely to report partaking in alternative activities than nonparticipants (*P*=.04). The most common reasons cited by nonparticipants were being unaware of the web-based program (24/56, 43%) despite a notification having been sent to the entire sample, lack of interest in the content (18/56, 32%), and technical issues (13/56, 23%), such as not owning or being able to fully use a computer. Both participants and nonparticipants were interested in a wide range of topics, with many being very particular about the topics they wished to access. Approximately half expressed willingness to pay for access; those who were willing to pay tended to have more years of education (*P*=.03).

**Conclusions:**

Our findings suggest a need for web-based activities for countering boredom and feelings of isolation. The main factors that influence the use, efficacy, and sustainability of online activities are access, motivational and need-fulfilling factors, and whether the activities are sufficiently tailored to individuals’ preferences and abilities. Challenges in substituting in-person services are promoting social relationships that are currently not sufficiently incorporated into most web-based programs, accommodating a wider range of topics, and increasing the accessibility of current programs to older adults, especially those who are homebound, both during and after the COVID-19 pandemic.

## Introduction

### Background

The COVID-19 pandemic has harsh implications for the quality of life of older adults. Stay-at-home orders, closure of senior centers, and restrictions on visits by friends and relatives have increased social isolation and loneliness in this population [1-3]. Some of the stressors experienced by older adults as a result of the COVID-19 pandemic are related to being confined to home, concern for the health and safety of family and friends, and boredom [4]. The latter has also been reported as an impact of quarantine restrictions [5]. Homebound older adults, who are unable to leave the house due to illness or other impairments, may be at higher risk of feeling lonely during the pandemic [6] and at other times [7,8].

Social isolation and loneliness increase older people’s risk of anxiety, depression [9], mortality [10], and dementia [11]. Activity and social engagement are important for psychological well-being [12], training of memory and executive function [13], greater happiness, and reduced mortality [14] of older adults.

Web-based technologies have been proposed as a way to address these issues while protecting older adults from COVID-19 infection [2,3]. These technologies are also cost-effective [15] and may benefit older adults in particular [16] because their social networks tend to be geographically less proximal [17].

Two categories of barriers have been identified concerning internet use among older adults: First, personal characteristics such as cognitive and physical impairments may limit their ability to use conventionally designed computer equipment [18,19]. Socioeconomic, educational, and cultural backgrounds influence older adults’ ability to access computers as well as the extent of their pre-existing knowledge of technology and their experience using it [20,21]. Living arrangements are also an important factor [22], as people who live alone are less likely to use the internet [20], and complete beginners and frail older adults require extensive support and assistance [23].

Second, attitudes of older adults toward the internet and technology, including computer anxiety [24] and concern about data security and privacy, can present obstacles [25,26]. Older adults may find themselves excluded from the “digital world” due to lack of knowledge and skill in the use of modern technologies, which stems from a lack of assistance and training as well as from technology designs that fail to consider their needs, knowledge, and background [27-29].

Studies investigating the impact of pilot social web-based interventions reported positive effects on loneliness [30,31]. Information and communication technologies were found to reduce depression [32], and smart technologies increased self-efficacy, empowerment, and confidence in using technology [33].

However, most of these studies are qualitative, with small sample sizes; thus, they may be less conclusive. In addition, the positive impact of these interventions on social support and connectedness has been found to be short in duration [34]. Although the frequency of internet use was associated with reduced loneliness, it did not impact perceptions of social isolation [35]. The type of web-based activity influences impact, as only social activities (eg, connecting with family and friends) were associated with decreased loneliness [36] and enhanced life satisfaction [37], whereas internet use for informational purposes or instrumental functions (eg, banking) were not. Recreational activities were the only type of activity with a significant correlation with older adults' well-being after controlling for background variables [38].

Research on web-based group activities for older adults is scarce. We found only 2 studies that examined the impact of web-based physical exercise activities in different settings, comparing individual and group training [39,40]. Baez et al [39] reported high usability of a group intervention but did not find a decrease in loneliness attributable to the group intervention itself. Importantly, the web-based group exercises in these two studies [39,40] included avatars in a virtual gym instead of live video communication, which may have impacted the perceived quality of social contact and hence loneliness.

### Aims of the Study

There is a need to further investigate the potential of web-based activities to enhance the well-being of older adults, particularly during the COVID-19 pandemic, where personal contact and interaction are severely restricted. Our specific research foci are (1) reasons older persons use or do not use web-based activities; (2) the effectiveness of various web-based activities in achieving user satisfaction and the use of alternative web-based activities; and (3) older persons’ activity preferences and their willingness to pay for web-based activities.

## Methods

### The Healthy Aging Web-Based Activity Program

An opportunity to examine these issues arose when we heard that after Healthy Aging, Ltd (*Beseva Bria*), a for-profit organization providing rehabilitation services to older persons, was forced to close its doors due to COVID-19 regulations, they started providing activities via the internet platform Zoom (Zoom Video Communications), which facilitates video group meetings. Web-based activities were initially offered free of charge when staff volunteered to provide older persons with activities and companionship after senior centers closed during the COVID-19 crisis. Thereafter, these Zoom meetings were offered for a small subscription fee.

Healthy Aging’s Zoom activities took place 5 days per week, with 3 activities per day, each lasting 30 minutes, between 10 AM and 11:30 AM. The first activity was always a type of exercise that could be performed while seated.

The other activities varied, including mindfulness, musical tai-chi, self-help, and lectures about topics such as world travel, history, health, and mental health. When deemed appropriate by Healthy Aging, some participants were invited to present lectures. Starting at 9:30 AM, some participants engaged in an informal chat via the same Zoom meeting. In the lectures and mindfulness activities, participants were encouraged to participate in discussions. The vast majority of the participants did not know each other, nor did they know the organization prior to connecting to the web-based program.

### Recruitment

After Healthy Aging agreed, we embarked on a pilot study to examine the utility of Zoom activities for the older population served by the organization. Ethical approval was obtained from the Institutional Review Board of Tel Aviv University.

**Figure 1:**
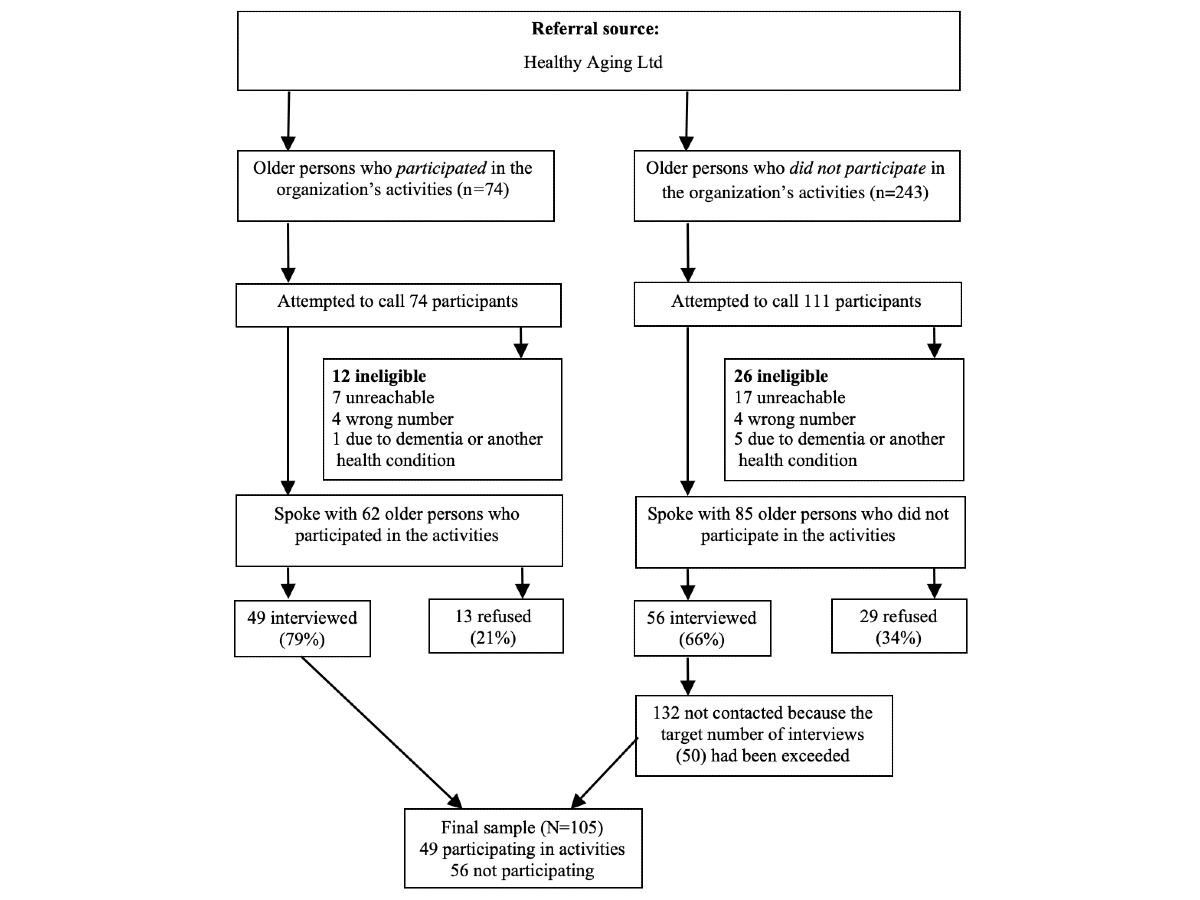
Participant recruitment process for the study.

Healthy Aging sent letters informing prospective interviewees of this study and offering them the opportunity to opt out of being contacted for it. Letters were sent to all persons who participated in the organization’s web-based activities or were offered the opportunity to participate. Healthy Aging then provided us with their contact information. The recruitment process is described in Figure 1. Although the percentage of people who declined to be interviewed was higher among people who did not participate in the activities (34%) than among activity participants (21%), this difference was not statistically significant (*P*=.08). We interviewed 49 participants and 56 nonparticipants.

### Assessments

Separate questionnaires were developed for participants and nonparticipants based on our research foci (Multimedia Appendix 1 and Multimedia Appendix 2, respectively). Interviews for both groups were conducted via telephone and lasted for approximately 20 minutes. Both interviews started with an explanation of the interview and a request for verbal informed consent. Both questionnaires included queries about the respondent’s demographic background. When someone other than the older person was the interviewee, questions were asked about the reason for this and the relationship of the interviewee to the older person.

The remainder of the questionnaire for activity participants included 37 closed- and open-ended questions, such as “How often have you participated in the activities?” rated on a scale of less than once a week, once or twice a week, three or four days a week, and every day. Comments offered in response to open-ended questions were transcribed. The topics covered were ease of using technology, frequency of participation, content of the activities, reasons for participation and their importance, satisfaction with particular activities, participation in alternative activities, desire for promotion of social relationships through the Zoom activities, and willingness to pay for web-based activities.

The questionnaire for respondents who did not participate in the activities included 25 similar questions, including their reasons for nonparticipation.

### Analytic Approach

Statistical analysis involved descriptive statistics using SPSS (IBM Corporation). The two groups were compared via *t* test for ordinal and interval data and via chi-square test for nominal-level data. When answers to 2 questions overlapped, such as “Why do you not participate in Healthy Aging’s online activities?” and “What changes or improvements in the online format would have motivated you to participate in particular activities?” the responses were combined. Open-ended responses were coded using an emergent coding strategy [41] whereby two research staff members read, coded, and categorized the responses independently and then revised the codes through discussion until agreement was reached. Their findings were reviewed by another staff member, and the main themes were ultimately agreed upon by all researchers. Open-ended responses are presented as quotations where they inform the quantitative findings.

## Results

### Demographics

As shown in Table 1, both groups had a mean age of approximately 74 years (participants, mean 74.3 years, SD 6.6; nonparticipants, mean 74.8 years, SD 8.7) and approximately 15 years of education (participants, mean 15.3 years, SD 4.2; nonparticipants, mean 15.2 years, SD 3.6). Over 80% of the respondents (89/105, 84.8%) were female. Over 90% (92/97, 95%) lived in their homes, and 60% (57/95) lived with a spouse. Demographic differences between those who participated in the web-based activities and those who did not were generally not significant. There was a trend for a larger percentage of nonparticipants (8/50, 16%) to require help in walking, compared to 2/47 participants (4%; *P*=.06) and in requiring help reaching places outside of walking distance (10/51 nonparticipants, 20%, vs 3/47 participants, 6%; *P*=.054). In 10/56 interviews (18%) with nonparticipants, someone other than the older person completed the interview, compared to 2% (1/49) in the case of participants (*P*=.008). In 13% (7/46) of interviews with nonparticipants who answered for themselves, the interviewer thought the respondent had some cognitive difficulties, compared to 2% (1/49) among participants (*P*=.04).

**Table 1:** Background variables of the study participants (N=105).

Variable		Participating in activities (n=49)	Not participating in activities (n=56)	Total sample (N=105)	Difference between groups (*P* value)	
Age (years), mean (SD)		74.3 (6.6)	74.8 (8.7)	74.6 (7.8)	.77	
Education (years), mean (SD)		15.3 (4.2)	15.2 (3.6)	15.3 (3.9)	.91	
Female gender, n (%)		43/49 (87.8)	46/56 (82.1)	88/105 (84.8)	.42	
Born in Israel, n (%)		26/47 (55.3)	32/52 (61.5)	58/99 (58.6)	.53	
Married (%)		27/46 (58.7)	28/51(54.9)	55/97 (56.7)	.71	
Residence (%)		43/45 (95.6)	49/52 (94.2)	92/97 (94.8)	.77	
**Living situation, n (%)**						.55
	With spouse	27/45 (60.0)	30/50 (60.0)	57/95 (60.0)		
	Alone	15/45 (33.3)	16/50 (32.0)	31/95 (32.6)		
	With caregiver	3/45 (6.7)	2/50 (4.0)	5/95 (5.3)		
	With other family member	0/50 (0.0)	2/50 (4.0)	2/95 (2.1)		
Can walk without help, n (%)		45/47 (95.7)	42/50 (84.0)	87/97 (89.7)	.06^a^	
Can reach places farther than walking distance without help, n (%)		44/47 (93.6)	41/ 51(80.4)	85/98 (86.7)	.054^b^	
Working, n (%)		9/46 (19.6)	16/52 (30.8)	25/98 (25.5)	.20	
Older person answered questions themselves, n (%)		48/49 (98.0)	46/56 (82.1)	94/105 (89.5)	.008^c^	
Impression of interviewer: cognitive impairment of person interviewed “Not at all,” n (%)		48/49 (98.0)	49/56 (87.5)	97/ 105 (92.4)	.04^d^	
Impression of interviewer: accurate information given, n (%)		49/49 (100.0)	53/56 (94.6)	102/105 (97.1)	.10	

^a^χ^2^_1_=3.6.

^b^χ^2^_1_=3.7.

^c^χ^2^_1_=7.0.

^d^χ^2^_1_=4.1.

### Feasibility: Extent of and Reasons for Participation and Nonparticipation

Of the 49 participants who participated in the activities, 27 (55%) reported participating in the activities every day, with 11 (22%) reporting participation 3 or 4 days per week; 37 (76%) had participated in the activities for over 20 days at the time of the interview.

Most of the 49 participants (34, 69%) were able to access Zoom on their own; however, 15 (31%) had difficulties, mostly in activating Zoom and starting the activities. These participants were helped by family members (9/49, 18%); by staff from Healthy Aging (4/49, 8%); by paid caregivers (1/49, 2%); or by a hired technician (1/49, 2%).

Respondents who participated in the activities rated enjoying the content of the activity as the most important motivator (mean score 4.4 on a scale of 1-5); as one participant commented, “This way you are exposed to interesting lectures and new people” [#162, age 74 years, female]. This was followed by maintaining a routine (mean score 3.6), as in “[It helps me] get up on time. …It provides me with a framework and routine” [#131, age 77 years, female]; enjoying the group and the presence of others (mean score 3.1); relief from loneliness (mean score 2.6), such as “Company during a time of loneliness” [#129, age 73 years, female]; and being motivated by family members or friends (mean score 1.8). One participant commented that the web-based activities helped her avoid loneliness and depression (#133, age 87 years, female).

Similar to the above ratings, the most common reasons for engaging in the web-based activities reported by participants were interest in the activities and relief from boredom for 30/49 (61%) (Table 2), followed by opportunity to exercise, access to activities from home, and maintaining a daily routine. Only 8/49 participants (16%) mentioned social activity or relief from loneliness as a reason for participating.

**Table 2:** Study participants’ reasons for participating (n=49) and not participating (n=56), n=105.^a^

Category and reason			Participants, n (%)
**Reasons for participating (n=49), 82 total responses**			
	**Motivation**		
		Interest in content/relieving boredom	30 (61)
		Providing exercise	15 (31)
		Maintaining a daily routine	11 (22)
		Social interest/relief of loneliness	8 (16)
		Activities appropriate for older persons	2 (4)
	**Access**		
		Activities accessible from home	14 (29)
		Convenient hours	2 (4)
**Reasons for not participating (n=56), 102 total responses**			
	**Access**		
		Never heard of Healthy Aging	24 (42)
		Technical issues^b^	13 (23)
		Inconvenient time or duration of the activities^c^	12 (21)
		Not willing to pay	3 (5)
	**Motivation**		
		Lack of interest in the content of the activities	18 (32)
		Participation in other activities/organizations	9 (16)
		The activities are designed for older persons (“I’m too young for these”)	5 (9)
	**Abilities**		
		Problems due to cognitive impairment^d^	7 (13)
		Hearing/vision problems^d^	4 (7)
	**Concerns**		
		Reluctance to take part in group activities	3 (5)
		Reluctance to participate in activities with a camera	1 (2)
		The organization seems too commercial	1 (2)

^a^Participants could provide multiple answers.

^b^Out of 13 participants, 8 (62%) did not know how to operate a computer/phone, 3 (23%) indicated they did not relate to technology and Zoom, and 2 (15%) had no computer.

^c^Out of 12 participants, 8 (67%) reported participating in other activities, 1 (8%) reported working, and 1 (8%) reported having no time due to caregiving for a spouse with dementia.

^d^Two persons reported cognitive reasons *and* hearing/vision problems.

Nonparticipants gave an average of 1.8 reasons for not participating (range 1-4, SD 0.9). The most common reasons were related to lack of access and awareness, such as not having heard of the activities sponsored by Healthy Aging (24/56, 43%) and technical issues (13/56, 23%), such as not owning a computer and inability or lack of knowledge of how to connect to Zoom. Motivational factors, such as lack of interest in the content of the offered activities, also played a role (Table 2), as did participants’ involvement in other activities (eg, “The activities offered are not as good as other activities I have and do” [#214, age 61 years, female]). Other reasons reported less often were cognitive and sensory problems and the perception that the activity was appropriate for an older group (Table 2). Respondents who felt the activity was appropriate for older persons expressed a preference for activities such as home repairs, yoga, and belly dancing. The average age of these respondents was 72.8 years (range 66-85 years), compared to 74.6 years for the full sample.

### Efficacy and Avenues for Upgrade: Participants’ Satisfaction With Web-Based Activities and Their Ideas for Improvement

As shown in Table 3, exercise was rated as the most satisfying activity (4.4 on a 5-point scale), and these activities were attended by most participants: “There is great diversity, every day, a different part of the body” [#110, age 75 years, female]. Mindfulness received the lowest ratings, with an average score of 3, denoting moderate satisfaction; one participant stated, “I do not relate to it” [#112, age 77 years, female]. The ordinal order of the levels of satisfaction for the different activities is roughly reflected in the attendance levels (Table 3), with the highest attendance at exercise and professional lecturer activities. The lowest attendance was reported for lectures provided by group members.

Out of the 49 activity participants, 28 (57%) expressed interest in continued participation in exercise activities after the COVID-19 pandemic was over, followed by interest in lectures, while other activities drew less interest. These reflect the same order of satisfaction with the activities (Table 3).

**Table 3:** Reported levels of satisfaction and desire to continue each type of activity offered by Healthy Aging after the COVID-19 pandemic (n=49).

Type of activity	Reported satisfaction levels per type of activity,^a^ mean (SD)	Number attending, n	Would like to continue the activity after the pandemic, n (%)
Physical exercise	4.4 (0.7)	44	28 (57)
Lecture^b^ (professional lecturer)	4.3 (1.0)	37	20 (41)
Lecture (lecturer from the participants’ group)	3.8 (1.0)	17	13 (27)
Self-care exercises (eg, head massage)	3.8 (1.4)	26	10 (20)
“Travel from the couch” lecture by a tour guide	3.7 (1.1)	25	14 (29)
Mindfulness	3.0 (1.5)	24	7 (14)

^a^1, not at all; 2, a little; 3, moderately satisfied; 4, satisfied; 5, very satisfied.

^b^Lectures included diverse topics, such as current art, memory, nutrition, the Bible, sexuality in old age, and history.

Participants indicated several ways through which their interest in participating in the activities could be increased, such as enhancing social contacts, enriching the content of activities, and improving technical and scheduling features. In terms of encouraging social contact, 42% (20/48; one participant did not answer this question) thought that social contact should be a goal of activities. In terms of the content of the activities, 33% of participants (16/49) viewed it as insufficiently interesting; in contrast, 2% (1/49) described the content as too complicated. Moreover, 2/49 participants (4%) considered the delivery to be too fast, and the same number requested more pictures and music in the presentations (2/49, 4%). Requests for additional types of content were especially common (13/49, 27%), as presented in Table 4, which includes requested content expressed by participants and by nonparticipants. The most popular specific topics were exercise, such as “I love Pilates” [#296, age 72 years, female]; culture, such as “lectures in museums” [#121, age 85 years, male]; music, such as “series of lectures about jazz” [#108, age 78 years, female]; art, such as “activities in the field of painting” [#224, age 86 years, male], and travel, such as “lectures on trips around the world” [#225, age 85 years, male]. However, this is not a perfect categorization, because many participants requested very specific activities, such as “play Remi,” “Nordic walking,” or “psychology of the brain.” Finally, 3 out of 49 participants (6%) mentioned problems with their computer, Zoom, or sound quality, and some requested simpler presentation formats, such as television. Out of the 49 activity participants, 10 (20%) sought programming at different times and during more time slots or recording of activities for later viewing.

**Table 4:** Preferred areas of interest of participants (n=49) and nonparticipants (n=56) in web-based activities (n=105).

Total (N=105), n (%)	Nonparticipants (n=56), n (%)	Participants (n=49), n (%)	Topic
33 (31.4)	13 (23.2)	20 (40.8)	Lectures—general
29 (27.6)	15 (26.8)	14 (28.6)	Exercise
25 (23.8)	10 (17.9)	15 (30.6)	Art/culture/music
12 (11.4)	6 (10.7)	6 (12.2)	Travel
6 (5.7)	2 (3.6)	4 (8.2)	History/philosophy
6 (5.7)	3 (5.4)	3 (6.1)	Coaching/body-mind
19 (18.1)	6 (10.7)	13 (26.5)	Other^a^

^a^Includes games/mind games/bridge (4 participants and 1 nonparticipant), literature (2 participants and 1 nonparticipant), science/technology (3 participants and 0 nonparticipants), education/social sciences (1 participant and 2 nonparticipants), religion (2 participants and 0 nonparticipants), food/cooking (0 participants and 2 nonparticipants), language study (1 participant and 1 nonparticipant), current affairs (1 participant and 0 nonparticipants), business (0 participants and 1 nonparticipant), gardening (1 participant and 0 nonparticipants), and wills (1 participant and 0 nonparticipants).

### Sustainability: Competing Activities and Willingness to Pay for Activities

Web-based activity participants were significantly more likely to report partaking in alternative activities (38/48, 79%) than nonparticipants (34/56, 61%; χ^2^_1_=4.1; *P*=.04). Of those participating in Healthy Aging’s web-based activities, 10/38 (21%) reported not participating in other activities. Of those not involved with Healthy Aging web-based activities, 22/56 (39%) reported not participating in any activities. Of these 22 individuals, 10 (45%) reported either cognitive, sensory, or technological problems. Alternative activities (Table 5) were accessed via television, such as a show with exercise instruction, or YouTube, Zoom, and websites of different organizations. Some participants reported that the activity was a continuation of a class they had taken in person prior to the COVID-19 pandemic. Sponsors of alternative activities varied, such as universities (eg, “Lectures on a film, and then [watching] the film” [#110, age 75 years, female]), museums (eg, “I have a subscription… and I listen to their lectures” [#120, age 63 years, female]), municipalities, synagogues (eg, “Prayer in the Synagogue via Zoom” [#103, age 79 years, female]), not-for-profit and for-profit organizations, and private individuals.

**Table 5:** Characteristics of the alternative activities engaged in by the study participants (n=73).

Activity or provider		Participants (n=39), n (%)	Nonparticipants (n=34), n (%)	Total (n=73), n (%)
**Platform**				
	Zoom	20 (51)	8 (24)	28 (38)
	Television	1 (3)	2 (6)	3 (4)
	YouTube	1 (3)	4 (12)	5 (7)
	Organization website	7 (18)	4 (12)	11 (15)
**Provider of activity**				
	University/museum	9 (23)	8 (24)	17 (23)
	Municipality	13 (33)	3 (9)	16 (22)
	Synagogue	1 (3)	0 (0)	1 (1)
	Not-for-profit organization	3 (8)	3 (9)	6 (8)
	Private person/organization	26 (67)	22 (65)	48 (66)

Of 47 respondents participating in web-based activities, 60% (n=28) said they were willing to pay for them, and 26% (n=12) were unwilling; meanwhile, 6 participants (13%) said “maybe,” and 1 (2%) said “I don’t know.” Among the interviewees who participated in the beginning of the program, 7 said they had stopped participating after Healthy Aging introduced a small subscription fee, while 14 reported that they were paying for participation at the time of the interview. Those who paid cited fairness and wanting or needing the service:

It’s worth it to me, it’s fair, and you get a lot for it

#103, age 79 years, female

I have no choice. If not for the activities, I'll be lost… So as not to be left alone [I choose to continue]

#133, age 87 years, female

Reasons for not wanting to pay varied, including the availability of alternatives (eg, “So far, I didn't feel the need to pay because there are a lot of options” [#138, age 68 years, female]); an ideological assertion that such services should be free at the time of a pandemic (eg, “Healthy Aging began [the program] nicely but then asked for money. I think it should continue for free until the end of COVID” [#121, age 85 years, male]); and “double billing”:

I used to participate in the exercise of Healthy Aging, but stopped when they required payment. I am not willing to pay because I am still paying a lot to my gym, despite not being able to go there.

#116, age 79 years, female

Out of the 56 nonparticipants, 20 (36%) indicated willingness to pay for Zoom activities; however, 12 (26%) responded in the negative. Although these percentages reflect less readiness to pay for such activities than reported by the activity participants, the difference is not statistically significant (*P*=.33). Those who were willing to pay tended to have more years of education (mean 15.8 years, SD 3.5) than those who said they would not or did not know if they would pay (mean 14.2 years, SD 3.6; *P*=.03).

## Discussion

### Principal Findings

Participants in Healthy Aging’s web-based activities reported very high levels of satisfaction with the exercise and lecture programs. Over 50% of the participants (28/49, 57.1%) wished to continue with the exercise sessions after the end of the COVID-19 pandemic, and over 40% (20/49, 40.8%) wished to continue with the lectures, citing the benefits of maintaining physical vitality, participating in interesting activities, and maintaining a daily routine. These results suggest that web-based activities are a viable substitute for the pre–COVID-19 activities of older adults. This finding aligns with Whitehead and Torossian’s [4] report that digital social contact was among the most commonly reported sources of joy or comfort for older adults during the COVID-19 pandemic. However, the results of our sample suggested insufficient provision of social contact and preventing loneliness—a benefit mentioned by only 16% of the participants (8/49). The lack of social cohesion may have also been reflected by the low attendance when lectures were presented by other participants. This finding supports previous studies that examined web-based exercise group activities and found no impact on loneliness [39,40,42] and research results indicating that older adults generally prefer face-to-face interactions [43,44]. The extent to and the conditions under which web-based activities can prevent loneliness should be explored in future research. However, the lack of social interaction in web-based activities is not unlike that at senior centers, which provide live lectures, concerts, or exercise classes; many participants arrive to these activities alone and leave without having had significant social interaction [45]. Strategies to promote social interaction both on the web and in person should be examined in future research.

### Conceptual Framework

Based on our findings, we have developed a conceptual framework that captures factors affecting the use, efficacy, and sustainability of web-based activities, as summarized in Table 6.

**Table 6:** Conceptual model: Factors affecting the use, efficacy, and sustainability of web-based activities.

Category		Factors
**Factors affecting use**		
	Access	Knowledge about program availability Sensory abilities (natural or modified) Technology access (personal or assisted) Sufficient funds to pay for subscription (real or perceived) Convenient hours and duration
	Concerns	Group activities Privacy: use of camera
**Factors affecting use, efficacy, and sustainability**		
	Fulfilling needs/motivational	Relieving boredom Providing physical activity Providing social interaction, relief of loneliness
	Tailoring activities to individuals’ preferences and abilities	Topic fit (interest) Level fit (difficulty match) Physical abilities Cognitive abilities Educational level Presentation fit (speed, timing, etc)

The main factors influencing the use of web-based resources were access and awareness. A sizable proportion of nonparticipants denied having heard of Healthy Aging’s web-based activities, despite having been notified about them by Healthy Aging. Further study should explore how to effectively promote and advertise web-based activities for older persons to ensure knowledge and comprehension of the activities by prospective users.

Another component of access was the technological challenges web-based activities pose for older persons, as reported by 31% (15/49) of the web-based activity participants—who nevertheless managed to participate—and 23% (13/56) of the nonparticipants, for whom technology was a complete barrier to participation. While this barrier might be overcome through the help of family members, caregivers, or staff from organizations, lack of such support remains a major barrier to internet and computer use by older persons [23]. Simplifying the process of use and adapting it to cognitive and sensory limitations may also aid in overcoming technological barriers. Indeed, sensory abilities, natural or modified, also affected participants’ access to web-based activities, as reported by 7% of nonparticipants (4/56). The extent to which compensation for these limitations can help disabled persons benefit from web-based activities needs to be examined.

Half of the nonparticipants (28/56, 50%) and 60% (28/47) of the participants said they would be willing to pay for web-based programming. Others were not inclined to pay for a range of reasons, including the availability of cost-free programs. A portion of this population may not be able to afford subscription fees.

Certain concerns emerged as factors that affected use. Some persons in our sample were reluctant to participate in activities involving a camera or a group. Such privacy concerns were previously reported as reasons for older adults’ hesitation to use the internet [25,26].

We identified physical activity, social interaction, and relief from boredom and loneliness as central motivational and needs-fulfilling factors that affected the use, efficacy, and sustainability of web-based activities. The preference for group activities, the importance of social interaction [40], and the opportunity to exercise were identified as motivators in previous research on in-person group exercises for older adults [46].

The final factor that we identified was tailoring activities to individuals’ preferences and abilities. The activities provided by Healthy Aging were an extension of its mission as a rehabilitation facility. Accordingly, programs focused on activities such as seated physical exercise, head massage, and stimulating lectures. However, for some respondents, these types of programs were considered to be relevant only to an older, frailer population.

Both participants and nonparticipants were interested in a wide range of topics, and many were very particular about favored topics, suggesting the commercial feasibility of developing wide-ranging lecture series; 50% of our sample were willing to pay for web-based activities. The suitability of the activity topics and content in our sample emerged as an important factor not previously identified in the literature.

The match between activity participation and participants’ cognitive and physical abilities as well as educational background proved to be critical. Cognitive or sensory problems interfered with the ability of some respondents to benefit from the web-based activities, consistent with prior literature [18,19]. Cognitive issues appeared to be more prevalent for nonparticipants, for whom both the percentage of interviews via third persons and the percentage of interviewees for whom interviewers suspected cognitive difficulty were significantly greater than in the case of the web-based activity participants.

### Limitations

This study is limited by its relatively small sample that is confined to one country and by its focus on a single rehabilitation organization that developed web-based activities for older persons during the COVID-19 pandemic. However, this narrow focus afforded us important insights into the experience of those who participated and those who declined as well as into the future potential of web-based programs for older persons.

### Conclusions

The closing of social clubs, senior centers, libraries, and gymnasiums due to COVID-19 critically affected the living experiences of older persons, who were instructed to limit social contact, stay at home, and decline visitors. Television and web-based activities were the main venues left to help older persons remain engaged and somewhat active. Our findings suggest that this alternative provides valuable substitute activities to a large portion of older persons who were required to limit outdoor activities due to the COVID-19 pandemic.

This study provides a preliminary investigation of the feasibility, efficacy, and sustainability of web-based activities among older persons. Each of these aspects of the study deserves a study of its own. Feasibility issues pertain to how to improve publicity and access to web-based programming. Efficacy relates to how to improve the match between the activities and the participants’ needs and wishes, as well as how to facilitate social interaction and to decrease loneliness. Finally, sustainability raises issues regarding funding, including participant contributions, to develop and present high quality activities that appeal to older persons.

In terms of enhancing access to web-based activities, training and support are needed for some older persons to access web-based programs [47,48]. In addition, it has been argued that improving older adults’ access to technology involves reducing the costs of computers, smartphones, and other equipment as well as adjusting their design to be more user-friendly to older adults [49]. As Seifert et al [49] aptly stated, older adults are impacted by “double exclusion”—social and digital—during the COVID-19 pandemic. These issues need to be explored in future research into the most effective means for increasing access to older populations with different types of limitations.

The results of this study have implications beyond the COVID-19 pandemic as a modality to address the needs of homebound older persons. The main challenges in substituting in-person services include the challenges of promoting social relationships within web-based platforms, accommodating a wider range of activities and contents on the web, and making current programs accessible to an underserved population that needs them, both through better marketing and via improving access to technology.

## Appendix

Multimedia Appendix 1 Questionnaire for older adults participating in the web-based activities.

Multimedia Appendix 2 Questionnaire for older adults who did not participate in the web-based activities.
