# Correction: Adequacy of Web-Based Activities as a Substitute for In-Person Activities for Older Persons During the COVID-19 Pandemic: Survey Study

**DOI:** 10.2196/27687

**Published:** 2021-02-16

**Authors:** Jiska Cohen-Mansfield, Aline Muff, Guy Meschiany, Shahar Lev-Ari

**Affiliations:** 1 Department of Health Promotion School of Public Health, Sackler Faculty of Medicine Tel Aviv University Tel Aviv Israel; 2 Minerva Center for the Interdisciplinary Study of End of Life Tel Aviv University Tel Aviv Israel; 3 Igor Orenstein Chair for the Study of Geriatrics Tel Aviv University Tel Aviv Israel

In “Adequacy of Web-Based Activities as a Substitute for In-Person Activities for Older Persons During the COVID-19 Pandemic: Survey Study” (J Med Internet Res 2021;23(1):e25848) the authors noted one error.

In the originally published article, the first sentence of the final paragraph of the section “Sustainability: Competing Activities and Willingness to Pay for Activities” read as follows:

Out of the 56 nonparticipants, 20 (36%) indicated willingness to pay for Zoom activities; however, 12 (26%) responded in the negative.

This was incorrect, and has been changed to:

Out of the 56 nonparticipants, 28 (50%) indicated willingness to pay for Zoom activities; however, 20 (36%) responded in the negative.

The correction will appear in the online version of the paper on the JMIR Publications website on February 16, 2021, together with the publication of this correction notice. Because this was made after submission to PubMed, PubMed Central, and other full-text repositories, the corrected article has also been resubmitted to those repositories.

