# Supplementary material for: Adequacy of Web-Based Activities as a Substitute for In-Person Activities for Older Persons During the COVID-19 Pandemic: Survey Study
Source: J Med Internet Res. 2021 Jan 22;23(1):e25848. doi: 10.2196/25848 (PMC7836908; doi:10.2196/25848)
Supplement: Multimedia Appendix 1 [file jmir_v23i1e25848_app1.docx]

Older Adults Participating in Online Activities Questionnaire

1. Participant number

Note: If a family member answers in the older person's place, ask questions 3 to 6. The questions should also be phrased in an adapted wording: for example, "Why is your family member not participating in the activities?" ^^[[1]](#footnote-1)^^

1. Why can’t the participant answer for himself/herself?
2. Would you be willing to respond on behalf of the participant?

1. Yes

2. No

5. What is your relationship to the participant?

1. Son
2. Daughter
3. Spouse
4. Other, please specify

6. What is the gender of the person answering the questionnaire?

1. Woman

2. Man

7. Interviewer number

8. Date of interview

9. How did you hear about the activities?

1. I received an email from the organizing team of Healthy Aging, Ltd.

2. Through the Jerusalem Municipality.

3. Through family member.

4. Through a friend.

5. Through the Healthy Aging, Ltd. website.

6. Other, please specify:

Details / comments:

10. Please describe how you accessed the technology (Zoom) to participate in the activity?

1. Independently, I know how to use it.

2. With the help of my care worker.

3. I was assisted by a family member/ friends/ neighbor.

4. I requested help from an Amdocs volunteer (organization that offers technological support).

5. I received assistance by the activities team (from Healthy Aging).

6. Other, please specify:

Details / comments:

11. How satisfied are you with Zoom as a medium for activities?

1. Not at all

2. Slightly

3. Medium

4. To a large extent

5. To a very large extent

Details/ comments:

12. Did you have problems connecting to or using Zoom?

1. Yes

2. No.

Please provide detail:

13. Degree of participation - How many days per week do you usually participate in activities?

1. Less than once a week

2. Once or twice a week

3. Three or four times a week

4. Participates every day

14. Degree of participation - For how many days have you participated in the activity so far?

1. 0

2. 1-2

3. 3-7

4. 7-10

5. 11-20

6. More than 20

Details / comments:

15. In which activities do you participate? (More than one answer can be specified.)

1. Morning exercise

2. Mindfulness

3. “Travel from the couch” – lecture by a tour-guide

4. Self-care (e.g., head massage)

5. Lecture from the group (by a fellow participant).

6. Lecture from an external party (by a professional presenter).

7. Other, please provide detail:

Details / comments:

16. If you responded affirmatively to watching lectures in the previous question, please specify which topics:

17. Did you lecture to the group?

1. Yes

2. No.

Please provide detail (was it successful? / were there any problems?)

18. What are the benefits of participating for you?

19. I will name a number of possible reasons for participation, and I would like you to rate how much each reason is relevant to you on a scale of: 1 = not at all relevant to me, 2 = slightly relevant to me, 3 = moderately, 4 = largely, 5 = very much.

1. Enjoyment of **group** activities and the company of other people.

2. Enjoyment of the specific **content** of the activity.

3. Relieving loneliness / enriching social life.

4. Maintaining a routine.

5. A family member motivated me to participate.

6. Other:

Details / comments:

20. Please rate your satisfaction with each of the following activities:

1 = not at all, 2 = a little, 3 = moderate, 4 = to a large extent, 5 = to a very large extent, 6 = not relevant / participant does not know the activity

1. Morning exercise
2. Mindfulness
3. “Travel from the couch” – lecture by a tour-guide
4. Self-care (e.g. head massage)
5. Lecture from the group (by a fellow-participant). On what topic?
6. Lecture from an external party (by a professional presenter). On what topic?
7. Other, please specify:

Details / comments:

21. Are there activities in which you choose not to participate? What was the reason?

1. Morning exercise

2. Mindfulness

3. “Travel from the couch” – lecture by a tour-guide

4. Self-care (e.g., head massage)

5. Lecture from the group (by a fellow-participant)

6. Lecture from an external party (by a professional presenter)

7. Other, please specify:

Details / comments:

22. What changes or improvements in the online format would have motivated you to participate in particular activities?

23. In addition to the online activity, would you be interested in participating in activities of the same type but in person?

1. Yes.

2. No.

3. Maybe.

4. Do not know.

Details / comments:

24. Have you participated in other activities on Zoom, in person, or in any other way (not of Healthy Aging, Ltd.) during the Corona period?

1. Yes.

2. No.

Details/ comments

If you have also participated in other activities, please answer the following questions (25-28):

25. In which organizations?

26. How are the activities transmitted?

1. WhatsApp

2. Zoom

3. Frontal

4. Other, detail:

Details / comments:

27. Please list the types of activities in which you participate and why:

28. Do you pay for these activities? If so, please specify amount:

29. If Healthy Aging encouraged establishing or developing friendships with other group members (for example, telephone or other meetings after the activities) as part of activities, would you be interested in that?

1. Yes.

2. No.

3. Maybe.

4. Do not know.

30. Please mark the activities that you would like to continue in the future (after COVID)?

1. Morning exercise

2. Mindfulness

3. A trip from the couch

4. Self-care (e.g., head massage)

5. Lecture from the group (by a fellow-participant). If so, on what topic?

6. Lecture from an external source (by a professional presenter). If so, on what topic?

7. Other, please specify:

Details / comments:

31. Please describe how you feel about the change requesting payment for participation in activities?

32. In the future, would you be willing to pay for participating in activities?

1. Yes.

2. No.

3. Maybe.

4. Do not know.

If so, what do you think is a reasonable amount to pay for participation?

33. What are the activity hours that are convenient for you?

1. Morning from 9:00

2. Morning but not before 10:00

3. In the afternoon 16: 30-17: 00

4. Evening 18: 00-19: 00

5. During various daily hours so I can participate when convenient

Details/ comments:

34. Do you have any further comments regarding the activities?

Demographic Characteristics [of participant in online activities]

35. Gender

1. Man
2. Woman

36. Year of birth:

37. Where were you born?

1. North Africa / Middle East
2. Israel
3. Western or Central Europe / USA / Canada
4. Russia / the former Soviet Union and Eastern Europe
5. South America
6. South Africa
7. Other

Details/ comments:

38. Marital status

1. Single
2. Married or in a relationship
3. Divorced or separated
4. Widow
5. Other, please specify:

39. Years of education - including elementary school, high school and college studies - please indicate only a number:

40. Where do you live?

1. In my house. With whom?
2. With family members
3. With friends
4. In sheltered housing
5. In a nursing home
6. Other, please specify:

41. If you answered "in my house" in the previous question, please specify with whom:

1. Alone

2. With a spouse

3. With another family member. Describe:

4. With a care worker

5. Other. Describe:

42. Are you able to walk?

1. Without help (except walking stick)
2. Need partial help (from another person or device)
3. Completely unable to walk on own.
4. No answer

Details / comments:

43. Are you able to reach places that are not within walking distance?

1. Does not need help (able to drive or travel alone by bus or taxi)
2. With partial help (need someone to help or accompany on the trip)
3. Unable to travel without arranging a special vehicle, such as an ambulance
4. No answer

Details / comments:

44. Do you work?

1. Yes, full time
2. Yes, part-time
3. No
4. No answer

Details / comments

45. If you work, was your work affected during Corona?

1. No, still working full time

2. Yes, my work changed to part-time

3. Yes, I do not work at all during this period

4. No answer.

Details / comments:

The following questions relate to the interviewer's perceptions during the interview and will be completed by the interviewer only.

46. To what extent do you think the interviewee understood the questions?

1. Not at all
2. To a small extent
3. Moderately
4. To a large extent
5. To a very large extent

Details / comments:

47. In your opinion, how motivated was the interviewee to answer the questions:

1. Not at all

2. To a small extent

3. Moderately

4. To a large extent

5. To a very large extent

Details / comments:

48. Have you identified cognitive problems: of memory, logical thinking, comprehension, etc.

1. Not at all

2. To a small extent

3. Moderately

4. To a large extent

5. To a very large extent

Details / comments:

49. Accuracy of the information provided

1. There is no reason to doubt the exact information

2. It seems that the interviewee reduced the severity of the problems and denied them

3. It seems that the interviewee exaggerated in describing the problems

4. It seems that the interviewee did not understand many of the questions

5. Other, please specify:

Details/ comments:

© Cohen-Mansfield, 2020

1. Questions were phrased in the second or third person depending upon whether the respondent was a study participant or someone answering on behalf of a study participant. [↑](#footnote-ref-1)
