# Supplementary material for: Adequacy of Web-Based Activities as a Substitute for In-Person Activities for Older Persons During the COVID-19 Pandemic: Survey Study
Source: J Med Internet Res. 2021 Jan 22;23(1):e25848. doi: 10.2196/25848 (PMC7836908; doi:10.2196/25848)
Supplement: Multimedia Appendix 2 [file jmir_v23i1e25848_app2.docx]

Nonparticipant in Online Activities Questionnaire

1. Participant number

Note: If a family member answers in the older person's name, add questions 3 to 6. The questions should also be read in an adapted wording: for example, "Why is your family member not participating in the activities?" ^^[[1]](#footnote-1)^^

2. Why can’t the participant answer for himself/herself?

3. Would you be willing to respond on behalf of the participant?

1. Yes

2. No

4. What is your relationship to the participant?

1. Son
2. Daughter
3. Spouse
4. Other, please specify:

5. What is the gender of the person answering the questionnaire?

1. Woman

2. Man

6. Have you heard of the activities?

1. Yes.

2. No.

Details / Comments:

7. If so, how did you hear about them?

1. I received an email from the organizing team of Healthy Aging, Ltd.

2. Through the Jerusalem Municipality.

3. Through a family member.

4. Through a friend.

5. Through the Healthy Aging, Ltd. website

6. Other, please specify:

Details / Comments:

8. Why do you not participate in Healthy Aging’s online activities (more than one answer can be marked)

1. I do not have a computer.

2. I do not have a camera on my computer.

3. I do not have a smart phone with a camera and internet connection.

4. I have a computer / smartphone, but I do not know how to use them.

5. The activities offered do not interest me.

6. I do not have time for them.

7. Due to cognitive inability. Do not understand what it is.

8. Difficulty concentrating.

9. Due to a hearing problem.

10. Due to a vision problem.

11. Other, please specify:

9. If the reason for non-participation is related to a technological difficulty, have you contacted one or more of the following sources:

1) The activities team?

1. Yes.

2. No.

What was the result of your request? / Why did you not request support?

2) The instructional videos on the Healthy Aging website?

1. Yes.

2. No.

What was the result of your request? / Why did you not request support?

3) Amdocs volunteers (organization that offers technological support)

1. Yes.

2. No.

What was the result of your request? / Why did you not request support?

4) Family members

1. Yes.

2. No.

What was the result of your request? / Why did you not request support?

5) Other, please specify

1. Yes.

2. No.

What was the result of your request? / Why did you not request support?

Details / Comments:

10. If the reason for non-participation is not related to a technological difficulty, which of the following changes would motivate you to participate in the activities?

1. Changing the program time

2. Change the duration of the activity

3. Voice activity only, such as a conference call / (no camera) on your phone or computer or tablet.

4. Vary the content of activities, please specify

5. Other, please detail:

Details / Comments:

11. Please rate the level of interest you have in each of the following activities:

1) Morning exercise

1. Not at all

2. Slightly

3. Moderately

4. To a large extent

5. To a very large extent

6. Not relevant / does not know the activity

Detail notes:

2) Mindfulness

1. Not at all

2. Slightly

3. Moderately

4. To a large extent

5. To a very large extent

6. Not relevant / does not know the activity

Detail notes:

3) A trip from the couch

1. Not at all

2. Slightly

3. Moderately

4. To a large extent

5. To a very large extent

6. Not relevant / does not know the activity

Detail notes:

4) Self-care (e.g., head massage)

1. Not at all

2. Slightly

3. Moderately

4. To a large extent

5. To a very large extent

6. Not relevant / does not know the activity

Detail notes:

5) Lecture from the group (by a fellow participant). If so, on what topic?

1. Not at all

2. slightly

3. Moderately

4. To a large extent

5. To a very large extent

6. Not relevant / does not know the activity

Detail notes:

6) Lecture from an external party (by a professional presenter). In which subject?

1. Not at all

2. slightly

3. Moderately

4. To a large extent

5. To a very large extent

6. Not relevant / does not know the activity

Detail notes:

7) Other, please specify:

1. Not at all

2. slightly

3. Moderately

4. To a large extent

5. To a very large extent

Detail notes:

12. Are there other types of activities in which you would like to participate?

1. Yes

2. No

If so, please specify which activities?

13. Would you be willing to pay for participating in an activity that interests you?

1. Yes.

2. No.

3. Maybe.

4. Do not know.

If so, what do you think is a reasonable amount for participation?

14. Have you participated in other activities (not from Healthy Aging, Ltd.) during the Corona period?

1. Yes.

2. No.

Detail notes:

If you have participated in other activities, please answer the following questions (13-16):

15. In which organizations?

16. How are the activities delivered:

1. WhatsApp

2. Zoom

3. In person

4. Other, detail:

17. Please list the types of activities in which you participate, and why?

18. Do you pay for these activities? If so, how much?

19. Please detail other topics related to the online activities you would like to comment on?

Demographic characteristics [of nonparticipant]

20. Gender

1. Man
2. Woman

21. Year of birth:

22. Where were you born?

1. North Africa / Middle East

2. Israel

3. Western or Central Europe / USA / Canada

4. Russia / the former Soviet Union and Eastern Europe

5. South America

6. South Africa

7. Other, detail: _____

Details / Comments:

23. Marital Status

1. Single

2. Married or in a relationship

3. Divorced or separated

4. Widow

5. Other, please specify:

24. Years of education - including elementary school, high school, and college studies - please indicate only a number:

25. Where do you live?

1. In my house

2. With family members

3. With friends

4. In sheltered housing

5. In a nursing home

6. Other, please specify:

Details / Comments:

26. If you answered in my house, please specify with whom?

1. Alone

2. With a spouse

3. With another family member, please detail

4. With a close care worker

5. Other, please detail:

Details / Comments:

27. Are you able to walk?

1. Without help (except walking stick)

2. Need partial help (from another person or device)

3. Completely unable to walk on own.

4. No answer

Details / Comments:

28. Are you able to reach places that are not within walking distance

1. Do not need help (able to drive or travel alone by bus or taxi)

2. With partial help (need someone to help or accompany on the trip)

3. Unable to travel without arranging a special vehicle, such as an ambulance

4. No answer

Detail notes:

29. Do you work?

1.Yes, full time

2. Yes, part-time

3. No

4. No answer

Details / Comments:

30. If you work, was your work affected by Corona?

1. No, still working full time

2.Yes, my work changed to part-time

3. Yes, I do not work at all during this period

4. No answer

Details / Comments:

The following questions relate to the interviewer's perceptions during the interview and will be completed by the interviewer only.

31. To what extent do you think the interviewee understood the questions?

1) Not at all

2) To a small extent

3) Moderately

4) To a large extent

5) To a very large extent

Details/ comments:

32. In your opinion, how motivated was the interviewee to answer the questions:

1. Not at all

2. To a small extent

3. Moderately

4. To a large extent

5. To a very large extent

Details / comments:

33. Have you identified cognitive problems: of memory, logical thinking, comprehension, etc.

1. Not at all

2. To a small extent

3. Moderately

4. To a large extent

5. To a very large extent

Details / comments:

34. Accuracy of the information provided

1. There is no reason to doubt the exact information

2. It seems that the interviewee reduced the severity of the problems and denied them

3. It seems that the interviewee exaggerated in describing the problems

4. It seems that the interviewee did not understand many of the questions

5. Other, please specify

Details / comments:

© Cohen-Mansfield, 2020

1. Questions were phrased in the second or third person depending upon whether the respondent was a study participant or someone answering on behalf of a study participant. [↑](#footnote-ref-1)
